# Analytical validation of an ultrasensitive multiplexed immunoassay for blood-based biomarkers GFAP, NF-L and tau

**DOI:** 10.21203/rs.3.rs-10616400/v1

**Published:** 2026-08-27

**Authors:** Catherine Demos, Nikhil Padmanabhan, Jermaine Brown, Taron Gorham, Daniel Romero, Rachel Cohen, Jason Abraham, Brian Ngo, Sol Rivera Velez, Allan Barnes, Jun Yang, David Graham, Allen D Everett, Frances Northington, Christopher Campbell, Martin Stengelin, Jacob Wohlstadter, George Sigal

**Affiliations:** Meso Scale Diagnostics, LLC; Meso Scale Diagnostics, LLC; Meso Scale Diagnostics, LLC; Meso Scale Diagnostics, LLC; Meso Scale Diagnostics, LLC; Meso Scale Diagnostics, LLC; Meso Scale Diagnostics, LLC; Meso Scale Diagnostics, LLC; Johns Hopkins All Children’s Hospital; Johns Hopkins School of Medicine; Johns Hopkins School of Medicine; Johns Hopkins All Children’s Hospital; Johns Hopkins School of Medicine; Johns Hopkins School of Medicine; Meso Scale Diagnostics, LLC; Meso Scale Diagnostics, LLC; Meso Scale Diagnostics, LLC; Meso Scale Diagnostics, LLC

**Keywords:** Biomarker, Glial fibrillary acidic protein, Neurofilament Light, Tau, immunoassay, analytical validation, precision, accuracy

## Abstract

**Background:**

Blood based biomarkers are increasingly used for non-invasive diagnostics, prognostics, stratification, and therapeutic monitoring, however clinical validation of new biomarkers is often limited by inconsistent assay methods. Assay developers can enable more reliable clinical studies by conducting analytical evaluation to characterize assay performance. In the context of brain injury and neurodegeneration, GFAP, NF-L and tau have emerged as potentially useful biomarkers for stratifying injury severity. Here we demonstrate analytical validation of the MSD^®^ S-PLEX Human Neurology Panel 1, an ultrasensitive multiplex immunoassay for GFAP, NF-L and total tau.

**Methods:**

Analytical validation testing consisted of multi-site, multi-lot precision, accuracy, stability, dynamic range, and interference. All testing was conducted with plasma, serum, and diluent-based control samples. The precision study was conducted at three sites: Meso Scale Diagnostics, LLC. (Rockville, MD), Johns Hopkins University (Baltimore, MD) and All Children’s Hospital (Tampa, FL). Three kit lots were tested at all three sites for a fully crossed study design. All runs contained an 8-point duplicate calibrator curve, duplicate sample measurements, and control samples. Data were analyzed at each site and then transmitted to MSD for combined analysis.

**Results:**

There were no significant differences between reagent-lot measurements. Intra-plate %CV averaged less than 10%. Between-site comparison of GFAP, NF-L and tau measurements revealed a small but significant difference in measurements across study sites. Accuracy reported that native samples recover within ± 20% of expected concentration through 20-fold dilution, and spiked samples recover within ± 20% through 256-fold dilution. No substances in a panel of potential interferents affected quantitation by more than ± 20% except for erythropoietin. Kits are shelf stable through 24 months, and GFAP, NF-L and tau in samples can withstand at least five freeze/thaw cycles, storage for 24 hours at 4°C, and storage for four hours at ambient temperature.

**Conclusions:**

The MSD S-PLEX^®^ Human Neurology Panel 1 has been analytically validated for research use biomarker studies measuring GFAP, NF-L and tau. Characterization of sources of variability have identified minor to moderate inter-site variability, but little to no inter-lot variability. Further work is needed to identify an international reference standard and to evaluate use in specific research contexts.

## Introduction

Blood-based biomarkers provide an avenue to improve disease diagnosis through inexpensive and non-invasive assay measurements. Biomarkers are also increasingly used in clinical studies as inclusion/exclusion criteria for selecting the appropriate populations, tracking drug pharmacokinetics and monitoring response to treatment^1^. Studies of new biomarkers, however, often suffer from poor reproducibility, especially in the neurology and neurodegeneration fields^2^. These differences can arise from underpowered clinical studies or overfitting of complex omics data, but also from analytical and pre-analytical factors affecting biomarker measurements such as poor assay reproducibility or a lack of certified reference materials for quantitation standardization. Assay developers can address some of these concerns by conducting a thorough analytical validation of assay methods to characterize potential sources of variability and inaccuracy prior to their use for biomarker discovery or clinical validation.

The proteins glial fibrillary acidic protein (GFAP), neurofilament light (NF-L) and tau have emerged as key biomarkers of brain injury and neurodegeneration, with applications in traumatic brain injury (TBI) and neurodegenerative diseases (NDD) including Alzheimer’s disease (AD) and related dementias (ADRDs). GFAP is a central nervous system-specific cytoskeletal protein that is released into biofluids, including cerebrospinal fluid (CSF) and blood, after brain injury^3^. NF-L is a neuron cytoskeletal protein that is released into CSF and blood upon axonal damage, making it a specific neuronal injury marker^4,5^. Tau is an intrinsically disordered microtubule-associated protein, closely associated pathologically with various neurodegenerative disorders such as AD and found in biofluids following brain injury in both children and adults^6,7^. GFAP, NF-L and tau are among the most promising biomarkers to depict depth and scale of injury, predict adverse outcomes, and indicate need for further intervention in hypoxic ischemic encephalopathy (HIE), the context under which this work was performed^8–11^. They are also relevant in the diagnosis of NDDs and for monitoring treatment response in the many ongoing clinical trials for novel ADRD therapeutics^12–14^. Despite the large body of work studying these biofluid biomarkers in brain injury and neurodegenerative disorders, there are few analytically validated assays available to support clinical research needs.

Here we present the analytical validation of a triplex assay panel – the MSD S-PLEX Neurology Panel 1 – for measuring GFAP, NF-L and tau. Our focus was on analytical validation to support future clinical research studies on the potential utility of these assays for assessing neurological risk in neonates with HIE, but the results support use in research studies for other neurological injuries and diseases. This assay requires no more than 5 μL of serum or plasma, quantitates over 5 logs of concentration range, and can be completed in less than 6 hours, or less than 4 hours using an accelerated protocol designed to provide faster time to result for the time-critical neonate HIE application. We will present data on multi-site and multi-lot precision, accuracy, real time kit stability, dynamic range, robustness to interferents, and sample stability. This study demonstrates utility of a fit-for-purpose, research grade, ultrasensitive, multiplexed immunoassay for use in biomarker research, clinical trial enrichment and as secondary endpoints for clinical trial outcomes.

## Methods

### 1. MULTI-ARRAY ^®^ Immunoassay Format

MSD multiplexed immunoassays measure targets with electrochemiluminescence (ECL) detection and a patterned capture antibody array^15^. The S-PLEX Neurology Panel 1 (cat. # K15639S) is a three- analyte ultrasensitive panel that boosts signal and sensitivity through the proprietary TURBO-TAG^®^ and TURBO-BOOST^®^ reagents^16–18^. Briefly, the assay protocol involves (i) coating the 96-well assay plate with capture antibodies, then adding (ii) blocking solution followed by samples, calibrator or controls, (iii) TURBO-BOOST-conjugated detection antibodies, (iv) enhance solution, (v) TURBO-TAG detection solution, (vi) read buffer and reading the plate, with incubation and washes between each addition. The protocol was carried out as described in the product insert, except that the assay incubation times and temperatures were adjusted (as outlined in Supplementary Table 1) to shorten the total assay time to four hours to provide a faster time to result for the neonate HIE application. Plates are read on an ECL plate reader (MESO^®^ SECTOR S 600 or MESO QuickPlex^®^ SQ 120). Kits, reagents and instruments are commercially available from MSD.

For quantitation of protein concentration, an eight-point calibration curve was run in duplicate on all plates and the signals for each sample were fit to a 1/Y2-weighted four-parameter logistic (4PL) fit. Sample concentrations for each analyte were calculated by back-fitting to the appropriate 4PL fit and correcting for dilution. Unless otherwise indicated, samples were run in duplicate and the average concentration is reported. All GFAP, NF-L and tau values are expressed in pg/mL and as mean ± standard deviation in text. The calibration standards used in the kit are purified full-length recombinant proteins. To maintain consistent quantitation across lots, new calibrator lots are assigned by anchoring to a reference lot.

### 2. Assay Sensitivity

The limits of quantitation were determined over multiple reagent lots. The guidelines within Clinical & Laboratory Standards Institute (CLSI) EP17-A2 “Evaluation of Detection Capability for Clinical Laboratory Measurement Procedures” were used for lower limit of quantitation (LLOQ), limit of detection (LOD), and limit of blank (LOB). There are no CLSI guidelines for upper limit of quantitation (ULOQ) determination, thus the protocol was adapted from the principles of EP17-A2. Samples bracketing the proposed limits of quantitation and detection were prepared by spiking individual purified calibrator materials into assay diluent, serially diluting as necessary, and storing in single-use aliquots to avoid repeated F/T cycles. Four levels each for ULOQ, LLOQ, and LOD were prepared, along with four independent lots of assay diluent to serve as LOB samples. Two operators conducted three plate runs each, one operator per independent reagent lot, over three calendar days. Samples were tested in quintuplicate alongside a standard duplicate 8-point calibration curve. (4 samples × 5 replicates/plate × 3 plates = 60 measurements/reagent lot for LOD and for LOB.)

LOB (the lowest concentration that can reliably be distinguished from a blank sample) and LOD (the lowest concentration that can reliably be distinguished from the LOB) were determined by ranking the 60 measurements per lot of the contrived LOB or LOD samples, respectively, and setting the values at the 95th percentiles within each lot. The LOB and LOD values for the different lots were compared and the highest determination was selected to ensure robustness across reagent lots.

ULOQ and LLOQ (the highest and lowest concentrations that can be reliably quantitated) were determined via the root mean square (RMS) model to calculate total error from the measured bias and precision. LLOQ for each lot was the lowest concentration for which total error was < 25%. The overall kit LLOQ was the highest LLOQ measured across the different lots. ULOQ was calculated in the same manner, however if the ULOQ in this experiment was determined to be above the top of curve (TOC), the TOC was set as the ULOQ.

### 3. Precision

Precision was established via a multi-site, multi-lot study designed according to the CLSI guideline EP05-A3 “Evaluation of Precision of Quantitative Measurement Procedures.” A 20×3×3 multi-site, multi-lot precision study was performed at three sites over approximately six months. The three sites were MSD in Gaithersburg, Maryland (MSD), Johns Hopkins University Hospital in Baltimore, Maryland (JHU), and Johns Hopkins All Children’s Hospital in St. Petersburg, Florida (ACH). Three reagent lots were independently produced and sufficient quantities of reagents for 20 plate runs per lot were sent to each site. 35 high-volume samples and 5 analytical controls were prepared at MSD in single use aliquots and shipped on dry ice to JHU and ACH. The samples included normal adult serum and plasma, analyte-spiked serum and plasma, normal umbilical cord serum and plasma, analyte-spiked umbilical cord serum and plasma, commercially-obtained clinical samples and sample pools from individuals with Alzheimer’s disease and multiple sclerosis, and challenge samples with human anti-mouse antibodies (HAMA+), anti-nuclear antibodies (ANA+), and hemolysis. Samples are described in full in Supplementary Table 2. Only samples with overall mean concentration within the quantitative range were included in precision estimates for a final tally of 27 samples. A total of 360 replicates were run for each sample and control (3 sites × 3 kit lots × 20 plate runs per kit × 2 replicates per run).

Prior to the start of the precision testing at each site, operators received training with the MSD reader and BioTek washer (Agilent Technologies, Santa Clara, CA, USA) as well as the accelerated protocol. Equipment was calibrated and logged and a preliminary checkout for the operators and equipment at each site was completed. During precision testing, each site tested up to two assay plates from two different reagent lots per day. Testing was conducted asynchronously across sites. Operators evaluated the calibration curves and quality control (QC) concentrations each day for confirmation of an acceptable run, and all raw data was transmitted to MSD for analysis. Runs were flagged if the calibrator curve Hill slope was outside of 0.9–1.1 and the R^2^ <0.9. Runs were also flagged if 3 or more of the QC samples recovered outside of the +/−30% range from the assigned concentration. Flagged runs were reviewed for sample or handling errors and were excluded only if attributable errors were found. Upon completion of testing, statistical analysis began with a global screen for aberrant points, drift and other anomalies for each sample/analyte combination by generating Levy-Jennings plots and measurement histograms. Grubb’s test using the MD68 statistic and median was performed for each dataset to calculate outliers. Subsequent analyses were performed both including and excluding outlier points, with the reported data including outliers. Groups of measurements between lots, sites, and runs were compared by ANOVA to test for statistical differences of means. Next, variance component analysis (VCA) was performed with R version 4.2.1, ‘VCA’ package version 1.4.5 to generate precision estimates describing the measurement variability attributed to run, lot, site, and residual, and to calculate reproducibility and total error. Inter-plate and intra-plate %CV(%CV=SD√×*100) were also calculated as a measure of precision. Total reproducibility for each QC sample was calculated independently for each lot, sample and analyte combination.

### 4. Real time kit stability

A real-time stability study was conducted through 24 months based on CLSI guideline EP25-A, “Evaluation of Stability of In Vitro Diagnostic Reagents.” Upon manufacturing of three independent reagent lots, sufficient reagents for all planned testing and 20% additional allowance for retests were stored according to manufacturer recommendations. Five QC samples were prepared as diluent spiked with purified analyte at levels spanning the detectable range, vialed into single-use aliquots and stored at ≤−70°C. The allowable acceptance criteria for QC measurements was ± 20% relative to their assigned value for the sample. 20 plate runs were performed at each time point of baseline (0), 12, 18 and 24 months. (20 plates × 2 replicates × 2 reagent lots over 4 timepoints each).

At each time point, QC sample measurements within or beyond the assigned concentration range were tallied. Intra-plate and inter-plate %CVs were calculated and compared to previous time points to detect changes in reproducibility. At the end of the 24-month timepoint, linear regression was performed for mean analyte results versus test time point for all samples. Slope statistics were examined to determine drift in measurements. If the linear regression slope was not statistically significant, the stability duration for that sample was taken as the maximum time point tested.

### 5. Sample Stability

Serum, plasma and QC samples underwent F/T and in-use stability testing. Native samples and purified analyte blends were aliquoted into single use vials and exposed to the following conditions: 1, 3 and 5 additional F/T cycles between room temperature and <−70°C, storage at + 4°C for 4 or 24 hours, and storage at room temperature (20–25°C) for 4 or 24 hours immediately prior to testing. All conditions for a particular sample were analyzed in duplicate on the same plate. Recovery to a baseline sample was calculated for each sample with allowable variance limits set to ± 20%.

### 6. Accuracy & Linearity

Accuracy was measured by dilution linearity, parallelism and spike recovery experiments.

A multi-stage approach to dilution linearity assessment was derived from CLSI EP06–2nd edition “Evaluation of Linearity in Quantitative Measurement Procedures.” First, commercially-obtained, pooled adult serum and plasma samples (n = 3 each) were spiked with purified analyte to levels in the high end of the assays’ quantifiable ranges. Samples were then diluted serially 2-fold a total of 8 times for a 256-fold range, and tested with 6 replicates per sample. Next, pooled neonatal serum samples were tested via linearity panel. High- and low-analyte level pools of HIE samples (n = 3 each) were obtained and a linearity panel was prepared from the pool by equal-volume mixing. The panel consisted of 25%/75%, 50%/50%, and 75%/25% mixtures of the high and low sample pools alongside 100% reference samples from each pool.

Finally, neonatal samples were tested individually to demonstrate parallelism and verify the claims of linearity established with spiked adult samples and pooled neonatal samples. A total of 64 individual serum samples from neonates with HIE (provided by Dr. Allen Everett) were tested at a dilution of 5x (minimum recommended dilution for injured neonatal serum), 10x and 20x. Further dilutions were not tested due to values falling below quantitation limits and due to the low sample volume available. A linear model with y-intercept fixed at 0 was used to analyze the relationship between measured concentration (dependent variable) and sample dilution. The deviation from linearity (measured concentration – predicted value) was plotted against predicted value.

Accuracy was also determined by spike recovery using large volume adult samples plus a mock sample (diluent) which were split and spiked with three levels of analyte. Spiked and unspiked aliquots were measured and recovery was calculated by (Spiked measurement – Unspiked measurement)/Spiked Diluent Concentration × 100. Spike recovery parameters were set at 80%–120% of assigned concentration.

### 7. Interfering substances

Common medical and laboratory substances such as antibiotics and analgesics were screened for interference with GFAP, NF-L and tau measurements according to the CLSI EP07 “Interference Testing in Clinical Chemistry” guideline. Paired-difference testing was employed to test samples with and without the interfering substance. Samples included native and analyte-spiked samples to determine interference at high and low concentrations of analyte. High concentrations in analyte-spiked samples were set according to the previously-established reference interval for concentrations of GFAP, NF-L and tau in neonates with severe HIE (Dietrick 2020, Li 2023, McGowan 2021). Each of the 15 potential interferents were dissolved in dimethyl sulfoxide (DMSO) (test) and spiked into serum and plasma samples in equal volume to DMSO only (control) at 3x clinically relevant concentrations (Supplementary Table 4). Eight replicates of each sample were tested and averaged to obtain mean concentrations for paired-difference analysis. The threshold for point estimate difference was set at 30%, and the 95% confidence interval was calculated.

### 8. Comparison of Original and Accelerated Assay Protocols

An accelerated protocol was developed, differeing from the product insert, to shorted time to results and better fit within a potential clinical laboratory workflow. Quantitation with the standard product insert versus accelerated protocols was compared by measuring a set of 62 K2EDTA plasma samples with known analyte concentrations spanning the quantifiable range, as well as the kit control samples. Correlation plots were generated for GFAP, NF-L and tau, and precision was determined by calculating intra-plate and inter-plate %CV.

### 9. Samples

Normal and diseased adult samples used in these studies were commercially obtained (BioIVT, Westbury, NY, USA). Normal human umbilical cord serum and plasma were commercially obtained (Anogen, Missassauga, ON, CA). Archived neonatal HIE serum samples were remnant clinical laboratory specimens collected with patient consent and Johns Hopkins IRB approval as part of NIH NICHD R01HD086058 “Adult biomarkers in neonatal brain injury and development”, and were provided by Drs. Northington and Everett for biomarker assessment and assay validation. All samples were de-identified prior to shipment.

## Results

### 1. Assay Range and Analytical Sensitivity

**Figure 1:**
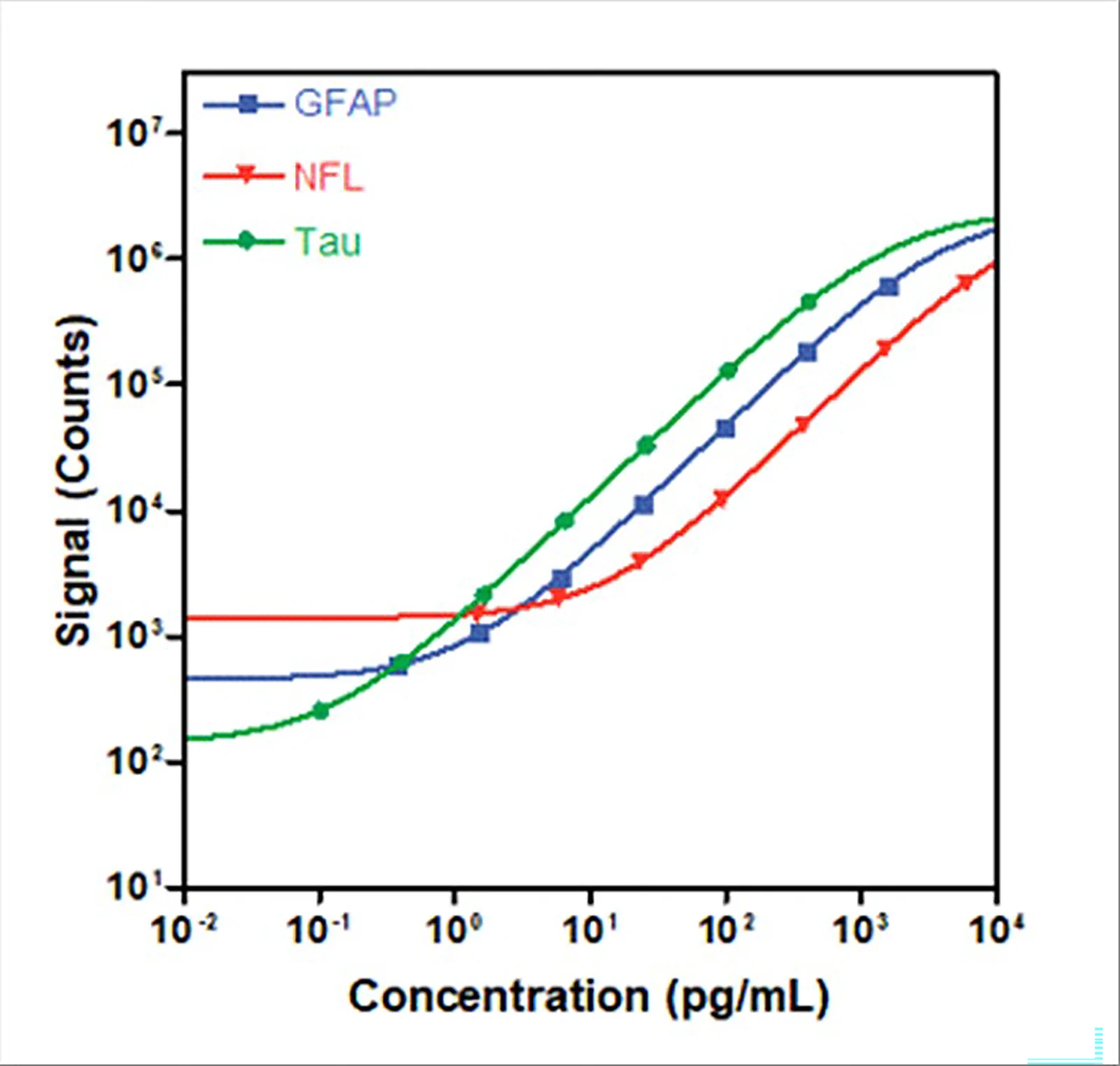
Representative calibration curves for GFAP, NF-L and Tau.

Figure 1 shows representative calibrator curves for the GFAP, NF-L and tau assays. The quantitation ranges (LLOQ – ULOQ) for undiluted samples are 2.3–4,230 pg/mL for GFAP, 7.7–10,030 pg/mL for NF-L and 0.69–735 pg/mL for tau (Table 1). Signals for the LLOQ for each analyte are at least 2-fold higher than the LOB signals. Reference studies using this panel have determined that normal adult serum and plasma samples range 11–62 pg/mL for GFAP, 21–350 pg/mL for NF-L and 0.5–73 pg/mL for tau (MSD S-PLEX Neurology Panel 1 Product Insert). Adult and neonatal brain-injured or diseased samples are elevated to 101–5,516 pg/mL, 2,339–13,577 pg/mL, and 319–3,447 pg/mL, respectively, the scale of which is highly dependent on injury severity^11,19^. Healthy neonatal blood was not tested due to availability, however normal umbilical cord serum for GFAP, NF-L and tau are 179 pg/mL, 117 pg/mL, and 0.6 pg/mL, respectively, and for plasma are 47 pg/mL, 84 pg/mL and 110 pg/mL, (Supplementary Tables 2 and 3). The recommended starting dilution for serum and plasma samples is 2-fold for adult samples and 5-fold for neonate samples. At these dilutions, the effective ULOQ is extended 2-fold and 5-fold for adult and neonate samples, respectively, while maintaining sufficient sensitivity to detect all three markers in healthy samples. Cases of extreme biomarker levels may be diluted further and maintain accuracy, as described in the Accuracy & Linearity results.

### 2. Multi-site precision of GFAP, NF-L and tau measurements

**Figure 2:**
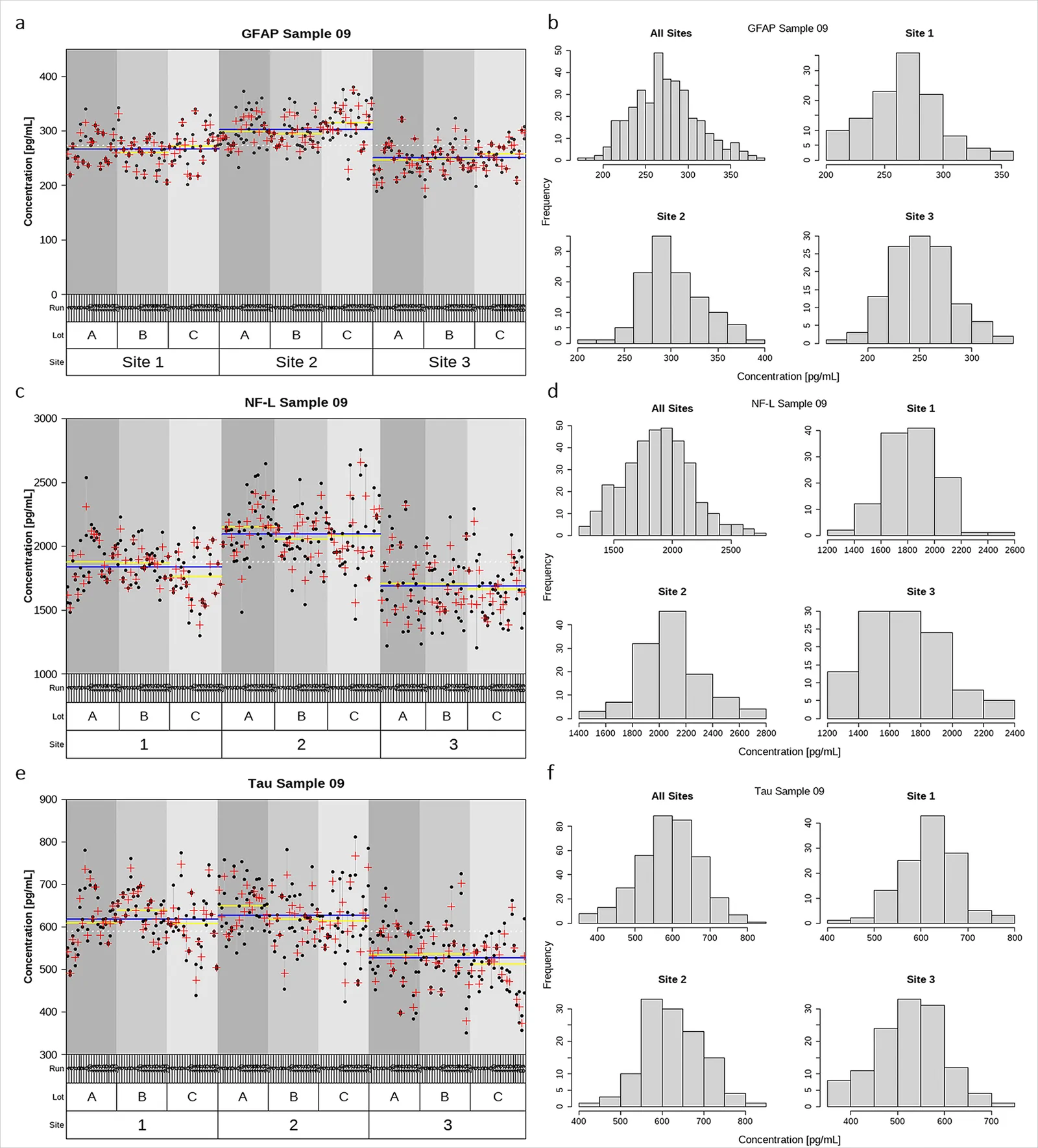
Levy-Jennings plots for Sample 09, a mid-level spiked umbilical cord serum sample. The plots for GFAP (a), NF-L (c), and tau (e) show individual measurements for each replicate (black points), average concentrations across plates (red cross), within-laboratory lot (yellow line), site (blue line) and overall mean (white dotted line). Measurement histograms for Sample 09, GFAP (b), NF-L (d), and tau (f) across each site and all sites combined showing normal distribution of measured concentrations across the 360 individual measurements for the sample for each assay across the three sites.

Figure 2a,c,e shows the Levy-Jennings plots and (2b,d,f) site-split histograms of GFAP, NF-L and tau measurements for Sample 09 as a representative of the visual analysis compiled for every sample/analyte combination. Assay-specific precision estimates were averaged across 27 samples and 4 QCs over the 60 plate runs per site to generate overall assay precision estimates, reported in full in Supplementary Table 3. Challenge samples that reported mean sample concentrations above the ULOQ or below the LLOQ (beyond the validated reportable range) were analyzed but excluded from aggregated assay precision estimates. Three samples (one analyte each) were identified as outliers, but ultimately included in the aggregated precision estimates. A total of 8 out of 180 NF-L runs were excluded due to failing calibrator and QC parameters. For individual sample errors, a total of 170 measurements were excluded (0.44% of all measurements). Total reproducibility %CV aggregated across the quantifiable range was 17.9% for GFAP, 19.4% for NF-L, and 16.8% for tau.

By VCA analysis, the largest contribution to variance for all three analytes was between-run variation at 11.7% CV for GFAP, 13.1% for NF-L and 12.8% for tau, and the smallest contribution to variance was reagent lot at 4.4% CV for GFAP, 3.5% for NF-L and 4.0% for tau. Differences in mean concentrations between sites were sometimes statistically significant, while lot-to-lot measurements within each site were not different, with each site reporting less than 5% variance for each analyte. Between-site variance was 8.5% for GFAP, 7.9% for NF-L and 5.3% for tau, and the residual within-run variance (repeatability) was 8.6% for GFAP, 10.2% for NF-L and 7.5% for tau. Within-laboratory (between-run) precision, calculated by VCA from one site with one operator and instrument across all three reagent lots and the five QC samples, was 14.1% for GFAP, 12.8% for NF-L and 13.4% for tau (Table 2), which is consistent with the overall between-run %CV reported across the entire study via VCA. All three reagent lots had equivalent within-laboratory precision estimates.

### 3. Accuracy & Linearity

**Figure 3:**
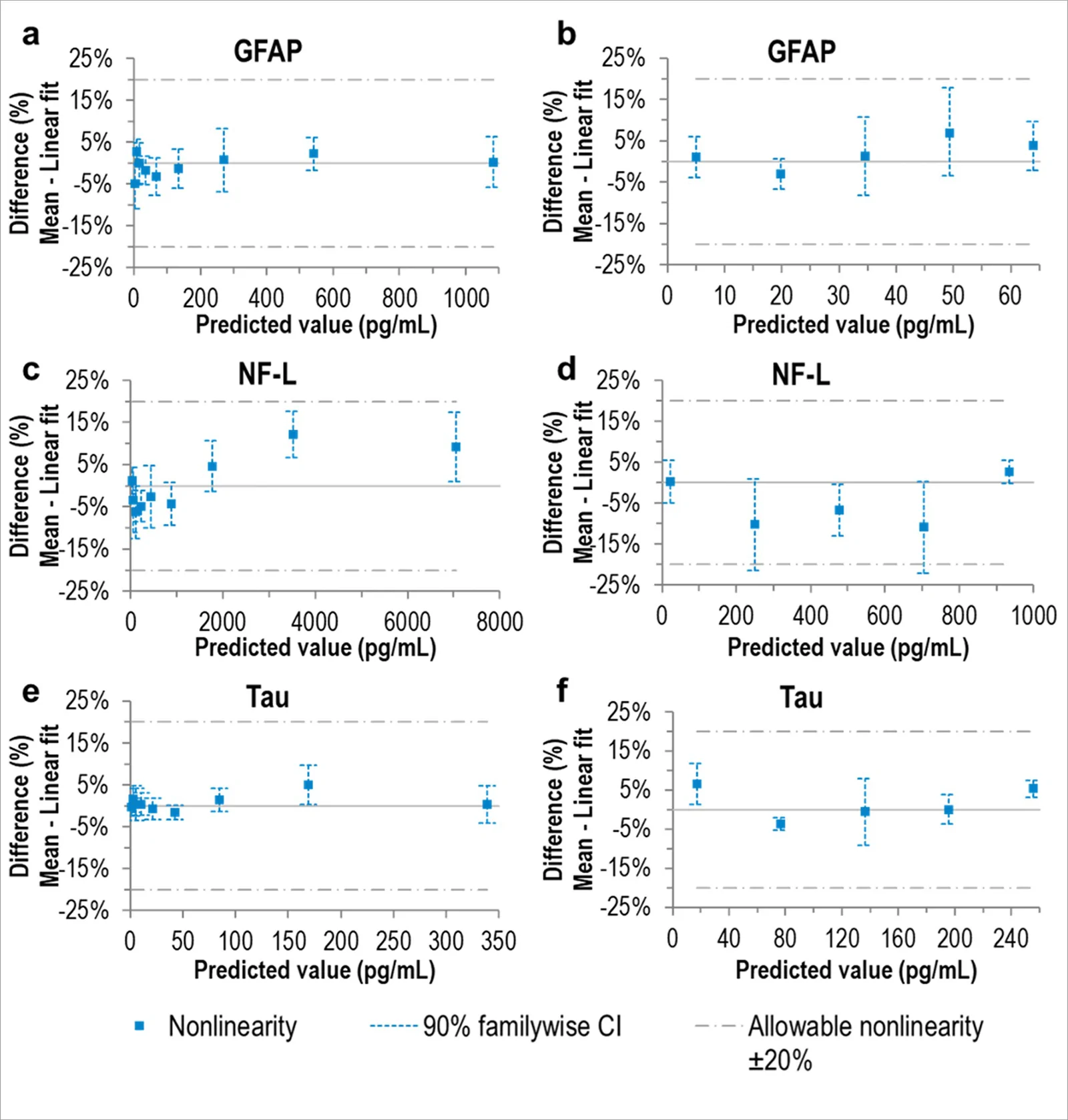
**a,c,e:** Representative plots of GFAP, NF-L and Tau dilution linearity for one sample each according to CLSI EP06 study design A1. Purified analyte was spiked into pooled adult serum and serially diluted in assay diluent. All values fell within 20% deviation from linearity (dashed lines). Data were analyzed using Analyse-it software. **b,d,f:** GFAP, NF-L and Tau dilution linearity according to CLSI EP06 study design B, which used a linearity panel produced by pooling neonatal HIE samples with high and low levels of analytes using equal-volume mixing. Representative data are shown for each analyte. All values fell within a 20% allowable deviation from linearity. Data were analyzed using Analyse-it software. Predicted value is calculated as the relative concentration to the high sample concentration, and % Deviation from Linear fit is calculated from the measured and predicted concentration. 95% CI calculated from n=6 replicates.

All three analytes fell within 20% deviation from linearity when spiked into serum and plasma and diluted serially to 256-fold (Representative plots for one sample shown in Fig. 3a,c,e, n = 6 samples were equivalent). Native analyte linearity tested by mixing high- and low-pools of known concentration analyte serum and plasma also fall within 20% deviation from linearity for all samples. Representative data from one panel shown in Fig. 3b,d,f.

**Figure 4:**
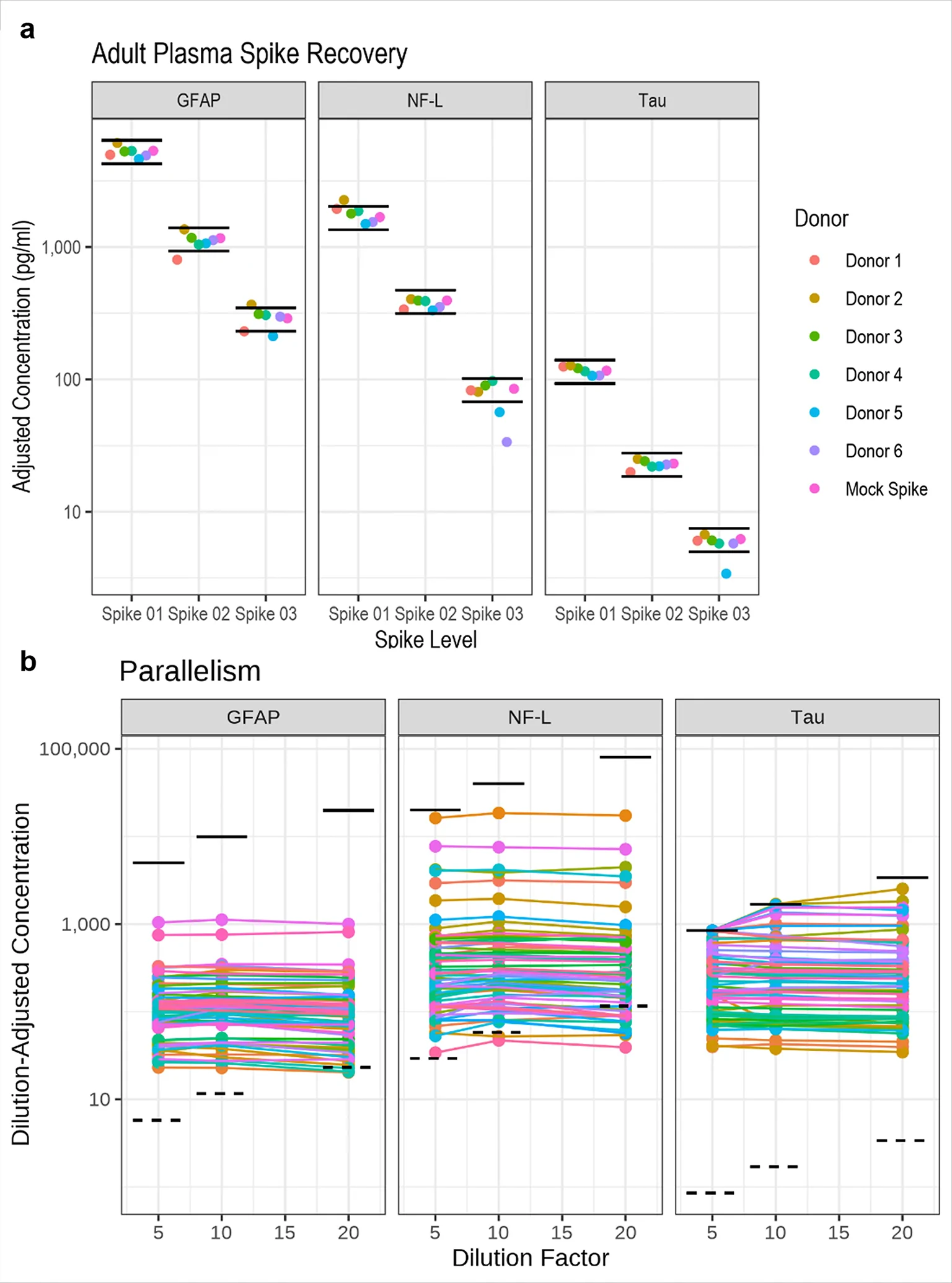
(a) Spike recovery testing of 6 individual adult plasma samples (commercially obtained) showing recovery of GFAP, NF-L, and Tau with respect to three Spike levels, and a Mock Spike sample (spiked diluent). Adjusted concentration is calculated by subtracting the unspiked measurement from the spiked measurement. Mock Spike sample shows measured expected concentration. Line segments indicate ±20% of expected concentration. (b) Parallelism testing of 64 individual neonatal HIE samples (cord serum). Samples with high endogenous levels of GFAP, NF-L, and Tau were diluted to 5-, 10-, and 20-fold before testing. Points are dilution-adjusted concentrations, each color is one sample. Line segments indicate dilution-adjusted LLOQ (dashed) and ULOQ (solid). Line and point colors represent independent samples.

Spike recovery of three spike levels in normal adult plasma samples was within 20% of the expected concentration for most measurements. Mean [Min-Max] percent recovery across all samples and spike levels for GFAP was 96.9% [68.8–126.7], for NF-L was 94.8% [38.5–134.4] and for tau was 96.3% [54.8–109.7] (Fig. 4a).

Parallelism of high endogenous analyte (Fig. 4b), shows the majority of individual samples fell within the allowable +/−20% deviation from linearity at both 10-fold and 20-fold dilution as compared to 5-fold. Some extremely high samples were above ULOQ at lower dilution levels (tau), and could not be quantified until diluted further.

### 4. Interfering substances

**Figure 5:**
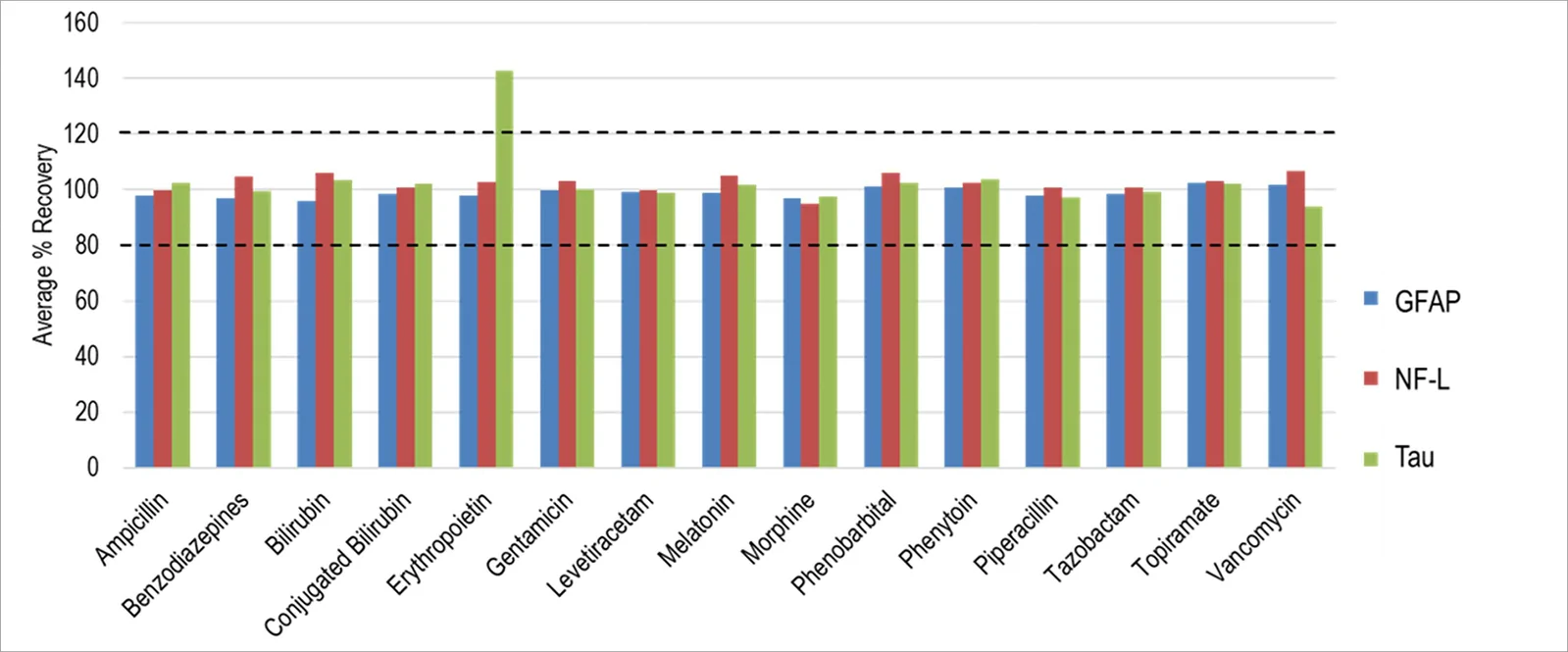
Common laboratory and medical substances were screened for interference with the neurology panel assays. EDTA plasma or Serum was either spiked with purified analyte near the upper end of the quantitative range or left non-spiked to test the lower end. Then, samples were spiked with either interferent dissolved in DMSO (test) or DMSO alone (control). Eight replicates of each were assayed, averaged then compared to non-spiked samples to calculate % Recovery. Average % Recovery is representative of high and low concentration samples.

Interferent testing conducted on both high and low biomarker concentration plasma and serum samples is summarized in Fig. 5. With the exception of erythropoietin, none of the tested substances affected GFAP, NF-L, or tau quantitation by more than 18%, with the confidence interval crossing zero for all cases. For erythropoietin, unusually high recovery was observed only for tau in unspiked (low analyte concentration) serum and plasma. This was not observed in spiked (high analyte concentration) serum or plasma, nor was it observed for NF-L or GFAP in the same samples. For the low analyte samples, the tau recovered at nearly double the expected concentrations, but the concentrations did not cross the cutoff value indicating neuroinjury^11^. Therefore, we conclude that there is no significant risk of biomarker measurement change due to these substances, when present at clinically relevant concentrations.

### 5. Real time stability of kit reagents including calibrators and controls

**Figure 6:**
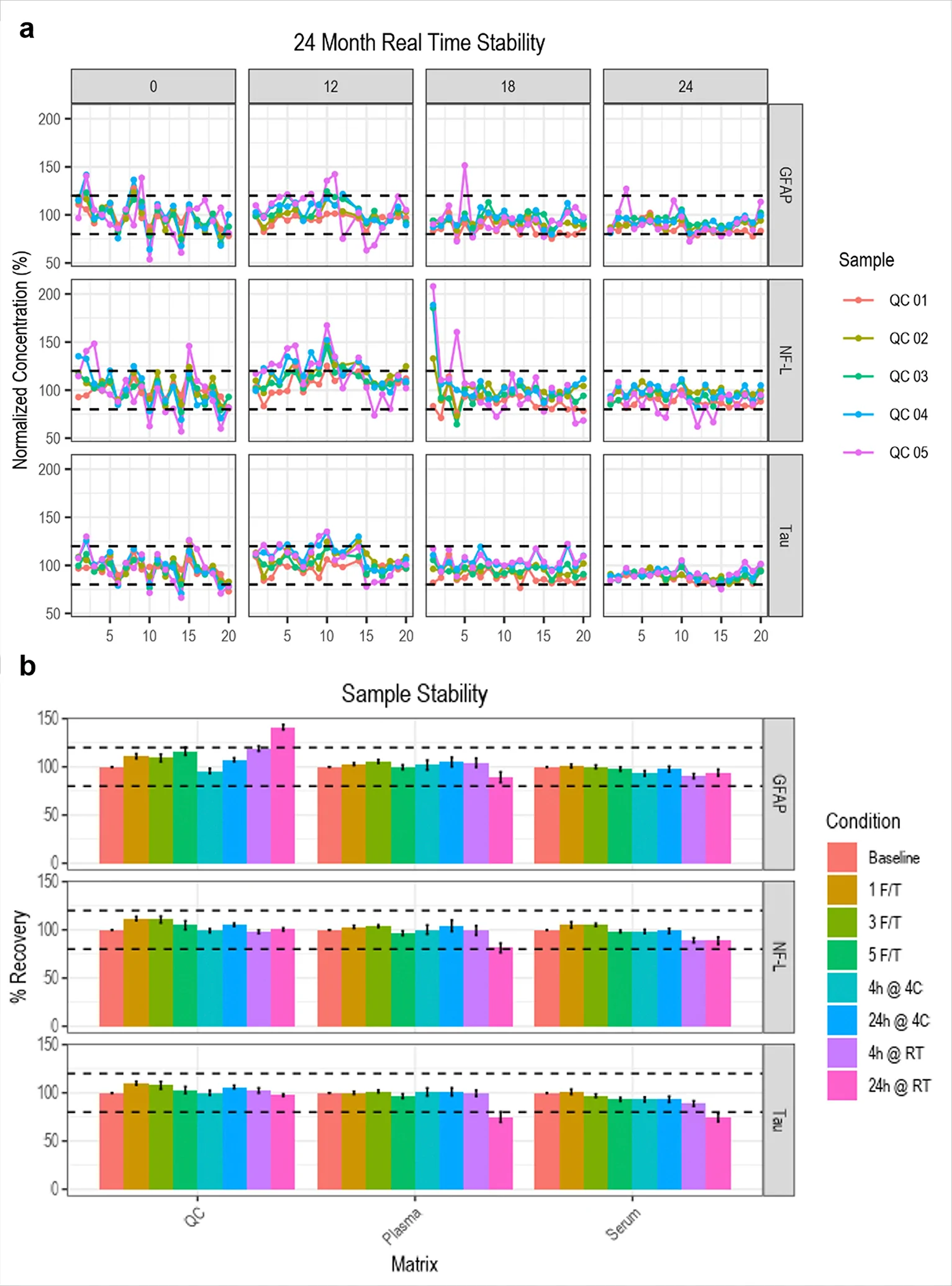
a) Real time stability of five QC samples spanning the analytical range for Lot A reagents over 0–24 months. Red dashed lines indicate +/−20% from target concentration. b) F/T and in-use sample stability. N=5 samples per matrix. Data normalized to baseline concentration within samples before calculating SE. Error bars indicate SE. Dashed lines indicate +/− 20% from baseline.

Figure 6a shows single lot results for 20 plate runs each across 0, 12, 18 and 24 months. One QC sample (QC 05) was below the LLOQ for GFAP and NF-L at baseline at the tested 1:5 dilution; data for this QC sample was excluded from the analysis for GFAP and NF-L. Linear regression was performed to measure any drift in QC sample quantitation, and to determine the estimated time at which QC sample quantitation would be expected to fall outside the allowed limits of variability (± 20% of nominal). For all three analytes, the estimated drift in mean measurements never exceeded the allowed limits of variability, so the shelf life was determined to be the last tested time point: 24 months following manufacture.

Median recovery to assigned concentration of the QC samples at each time-point ranges from 89.1%–103.2% for all analytes. The 25th to 75th percentile of control recovery is fully contained within the target 80%–120% range at each time point.

### 6. Sample stability

Serum, plasma, and QC samples were subjected to in-use and additional F/T conditions to assess analyte stability. The measured analyte concentrations after each storage condition, presented as normalized concentration relative to the baseline concentration, is shown in Fig. 6b. The only condition which did not fall within the target 80–120% recovery range for any analyte or sample matrix was 24 hours storage at RT, indicating prolonged time at this temperature may subject the analyte to degradation in sample matrix.

### 7. Shortened protocol comparison

**Figure 7:**
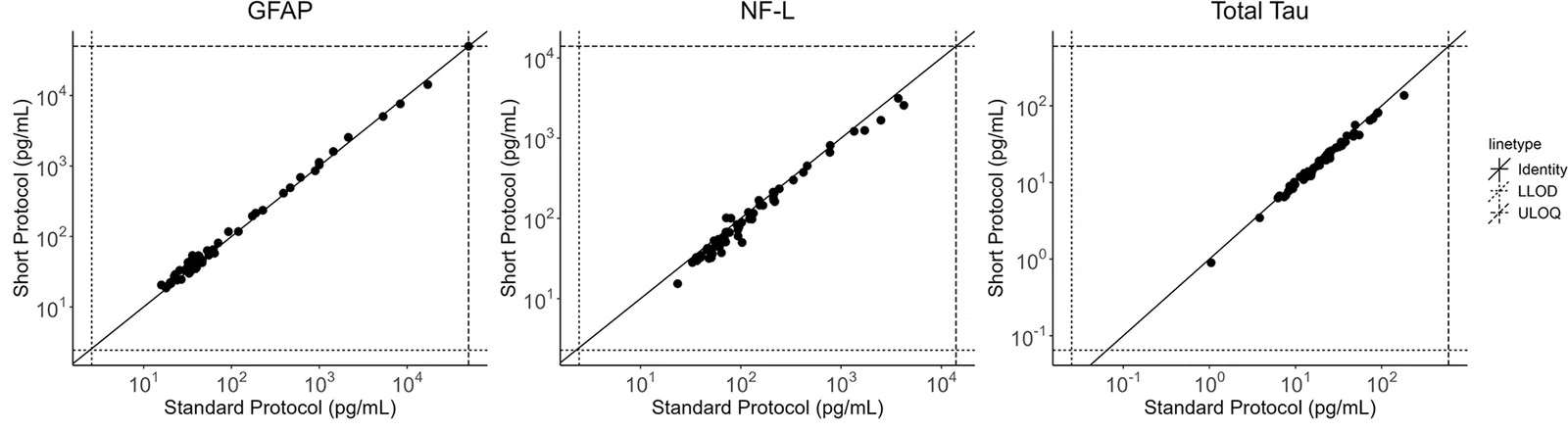
Concordance of neonatal HIE sample measurements on the neurology panel between the Standard Protocol and the Shortened Protocol plotted against an identity line (solid). Dashed lines indicate limits of quantitation.

As the assay protocol presented here differs from the commercial product insert protocol, a head-to-head comparison of the standard and shortened protocols was performed using remnant plasma samples containing a wide range of native GFAP, NF-L and tau concentrations. The shortened protocol quantitated samples equivalently to the standard protocol. As shown in Fig. 7, measured concentrations were highly correlated (r > 0.99). All samples were quantifiable for all assays. Weighted residuals for nearly all samples (61 of 62) were within the target range of ± 20%. One sample had a residual value of 35% for GFAP and a residual value of 21% for tau. There was no bias in weighted residuals across the range of sample concentrations. Weighted residuals were approximately normally distributed.

In order to estimate the intra-run CV for the GFAP, NF-L and tau assays, we analyzed the intra-plate %CV for the 62 samples, which were run in duplicate. CV of duplicates for the shortened protocol tended to be lower than for the standard protocol, although this difference was not statistically significant. Mean intra-run CVs (95% confidence interval) for the standard protocol were 5.4% (CI = 4.3%–6.6%) for GFAP, 9.8% (CI = 7.4%–12.1%) for NF-L, and 5.8% (CI = 4.5%–7.1%) for tau. Intra-run CVs for the shortened protocol were 4.0% (CI = 3.2%–4.9%) for GFAP, 8.3 (CI = 6.2%–10.4%) for NF-L, and 2.8% (CI = 2.1%–3.5%) for tau.

## Discussion

Biomarker research for neuroinjury and neurodegeneration has experienced rapid acceleration in recent years, benefitting from the use of advanced imaging techniques as a gold standard, the availability of large-scale biorepositories of test samples, and improvements in antibody reagents and assay methods. Clinical trials are increasingly relying on blood-based biomarkers as surrogate and secondary endpoints in an effort to allow for less invasive and less expensive diagnostics, prognostics, and treatment monitoring. However, comparison of results across studies and assay methods remains difficult due to a lack of international quantitation standards and the use of research grade assays without robust analytical validation. To address the need for assays that can provide reproducible quantitation across neurology biomarker studies, we analytically validated a research-use-only (RUO) triplex assay panel measuring GFAP, NF-L and tau – the MSD S-PLEX Neurology Panel 1 – according to the Clinical & Laboratory Standards Institute (CLSI) guidelines. Though this analytical validation was undertaken in the context of HIE, the biomarkers themselves are broadly applicable to other neurology research areas such as traumatic brain injury, neurodegeneration such as Alzheimer’s disease (AD) and AD-related dementias (ADRD) and infectious disease.

At the recommended sample dilutions (2-fold for adult samples, 5-fold for neonate samples), the analytical sensitivity and dynamic range of each of the three assays in this multiplex panel are sufficient to detect biomarker levels in healthy normal serum and plasma samples and most injured or diseased samples. In rare, severe injury cases, biomarker levels may be above the quantitation limits at these dilutions, however the quantitation limits are inclusive of the relevant suggested clinical cutoff levels^11,19,20^. The required assay volume for adult and neonate samples is 12.5μL or 5μL per replicate, respectively, minimizing the consumption of precious study samples.

We evaluated assay precision in two stages: a single-site, single-lot precision study to evaluate within-lab and between-operator reproducibility, followed by a multi-site, multi-lot precision study to evaluate between-laboratory and between-reagent lot reproducibility. The main finding across both studies is that the precision error is less than 20% overall for all three assays across all runs, indicating that reproducible results can be obtained from a trained operator at any site, at any time, and using any reagent lot. Specifically, the lot-to-lot error is less than 5% across three independent reagent lots, validating the consistency of calibrator performance across lots, and implying longevity for long-term studies such as those investigating neurodegeneration, downstream impact of brain injury, and exploratory or supplemental endpoints in long-running clinical trials. Similarly, the between-site error was less than 9% for all three biomarkers as determined by ANOVA. The greatest site-wise differences in measurements occurred at the extreme ends of the reportable range, supporting other evidence that the clinically-relevant concentrations within the linear range of the assay are less susceptible to site bias. The precision study also included challenge samples such as those with hemolysis and anti-nuclear antibodies, and no significant change in precision was observed. Increased relative imprecision was observed for samples at or near LLOQ, as expected due to signal-to-noise constraints. Critically, relevant clinical cutpoints lie above the region of inflated imprecision, and the precision estimates within the reportable range meet the EP05 guideline expectations.

One limitation of this study is the absence of internationally recognized reference materials against which absolute accuracy for GFAP, NF-L and tau quantitation can by established. There are no current CLSI guidelines to determine accuracy, and no international reference standard materials against which to establish absolute accuracy for quantitating GFAP, NF-L and tau. Despite the lack of such materials, the reference standards used herein consist of purified full-length recombinant proteins characterized by mass spectrometry. In the absence of certified standards, accuracy was evaluated using complementary approaches including dilution linearity, parallelism in native clinical samples, and spike recovery across multiple concentration ranges. Dilution linearity was examined in three study designs. First, plasma and serum samples spiked near the upper limit of quantitation show linearity from neat through 256-fold dilutions, with mean recovery within ± 10% for all analytes. This supports claims of linearity and allows for extreme values to be accurately quantitated using high dilutions. Second, a linearity panel was prepared by equal-volume mixing of a high-analyte pool and a low-analyte pool of HIE samples. Demonstrating that linearity claims hold with the native analyte, the linearity panel also recovered within ± 10% of the expected concentration. Finally, individual injured neonatal plasma samples were diluted 5-, 10-, and 20-fold. Spike recovery was also employed on adult serum and plasma, and the target spike concentration added to the measured native concentration was calculated as the expected concentration. Across these designs, measured concentrations were consistently within ± 20% of expected values throughout the validated reportable range. Instances of larger variability were observed primarily at concentrations near the lower limit of quantitation, where increased relative imprecision is anticipated due to signal-to-noise constraints inherent to ultrasensitive immunoassays. Importantly, concentrations corresponding to clinically relevant injury thresholds fell well within ranges demonstrating robust precision and linearity, supporting the suitability of the assay for biomarker research applications.

A critical component to biomarker measurements in real world applications is the contribution of potential interferents such as analgesics, antibiotics, and common laboratory substances. We observed measurable interference from erythropoietin at low endogenous tau concentrations, resulting in increased apparent recovery in unspiked samples. This effect was not observed at higher tau concentrations, nor for GFAP or NF-L, and did not result in misclassification relative to published neuroinjury thresholds. Nonetheless, these findings highlight the importance of considering patient treatment context and evaluating potential assay interference in specific clinical trial settings. Overall, the data support reliable and reproducible quantitation across the validated dynamic range, with analytical behavior consistent with expectations at the extremes of measurement. With the advent of monoclonal antibody treatments against tau currently in various stages of clinical trials^21^, it is important for investigators to determine potential interference from their particular monoclonal tau antibody agent. Specific interference and cross reactivity of monoclonal tau antibody therapeutics should be evaluated on a case-by-case basis in advance of any study using any tau immunoassay as a surrogate or secondary endpoint.

Reagent stability and analyte stability in samples are both factors to consider prior to beginning any study. The kit reagents show no drift in quantitation of control samples through 24 months of shelf life, ensuring consistent quantitation between, for example, initial measurements and follow up studies. Further, preanalytical factors in sample handling can affect many assays if the target analytes are not stable or succumb to matrix effects. Many retrospective studies are performed on subaliquot samples, introducing potential variance as it relates to collection methods or freeze/thaw cycles. Here we show in-use stability of samples at 4°C and RT, and F/T stability through 5 F/T cycles. All three analytes are stable in serum, plasma and assay diluent through 1, 3 and 5 F/T cycles, showing no analyte degradation from baseline. GFAP and NF-L are stable through 24 hours at 4°C and RT, and tau is stable through 24 hours at 4°C and 4 hours at RT, suggesting highly stable analyte and consistent quantitation.

The shortened protocol designed for this study differs from the standard product insert protocol by shortening certain incubation times and using a heated incubated shaker at 27°C to allow specific reactions to reach equilibrium earlier. This was developed with faster time to results as a goal of the project. Comparison between the two protocols in a head to head test show equivalent quantification and precision, although the shorter protocol lost some sensitivity, as evidenced by higher limits of quantification. Despite the lower sensitivity, the dynamic range was sufficient for the application of identifying neural injury, and all tested samples remained well within the quantifiable range.

## Conclusions

The studies presented herein represent analytical validation of an ultrasensitive, multiplexed immunoassay for the neurology biomarkers GFAP, NF-L and tau. This assay is sufficiently sensitive to detect these analytes in normal serum and plasma and has a large dynamic range to accommodate elevated levels found in target neurological disorders. The panel has demonstrated excellent precision, quantifying samples equivalently across reagent lots, operators, and laboratories, and is accurate across high level dilutions, high and low biomarker concentrations, and with native and recombinant analyte. The assay reagents are stable through at least a 2-year shelf life and the analytes are shown to be robust to preanalytical factors.

## Supplementary Material

This is a list of supplementary files associated with this preprint. Click to download.

• SupplementaryTables.docx

Supplementary Table 4

Substances tested for interference with assay measurements, and the final in-sample concentration tested.

## Figures and Tables

**Table 1:** Limits of detection and quantitation for neat samples.

Conc. (pg/mL)	LOB	LOD	LLOQ	ULOQ
GFAP	1.7	2.1	2.3	4,230
NF-L	3.2	5.0	7.7	10,030
Tau	0.26	0.32	0.69	735

**Table 2:** Within-Laboratory Precision (%CV)

Lot	Sample	GFAP	NF-L	Tau
Lot A	QC 01	11.2	9.7	12.1
	QC 02	14.1	13.4	14.1
	QC 03	12.9	14.0	12.8
	QC 04	10.7	13.2	12.3
	QC 05	27.1*	18.1	15.3
Lot A Average		15.2	13.7	13.3
Lot B	QC 01	9.3	9.2	9.9
	QC 02	10.8	11.3	13.6
	QC 03	12.7	12.4	12.2
	QC 04	19.1	14.4	15.1
	QC 05	17.8	18.6	17.1
Lot B Average		13.9	13.2	13.6
Lot C	QC 01	11.7	8.2	10.9
	QC 02	9.3	10.5	10.3
	QC 03	10.2	8.5	12.0
	QC 04	17.8	15.1	17.3
	QC 05	17.3	16.1	16.1
Lot C Average		13.2	11.7	13.3
Overall Average		**14.1**	**12.8**	**13.4**

## Data Availability

All data generated or analyzed during this study are included in this published article [and its supplementary information files].

## Abbreviations

GFAP: Glial Fibrillary Acidic Protein
NF: L–Neurofilament Light
HIE: Hypoxic ischemic encephalopath
TBI: Traumatic brain injury
NDD: Neurodegenerative diseases
AD: Alzheimer’s disease (AD) and related dementia (ADRD)
CSF: Cerebrospinal fluid
ECL: Electrochemiluminescence
CLSI: Clinical Laboratory Standards Institute
LOB: Limit of Blank
LOD: Limit of Detection
LLOQ: Lower Limit of Quantitation
ULOQ: Upper Limit of Quantitation
TOC: Top of Curve
CV: Coefficient of variance
VCA: Variance component analysis
DMSO: Dimethyl sulfoxide
